# A Case of Capillary Leak Syndrome and Intestinal Ischemia Caused by Rheumatoid Vasculitis

**DOI:** 10.7759/cureus.33404

**Published:** 2023-01-05

**Authors:** Atsuki Katsube, Junya Ohara, Yuta Horinishi, Chiaki Sano, Ryuichi Ohta

**Affiliations:** 1 Family Medicine, Shimane University Faculty of Medicine, Izumo, JPN; 2 Family Medicine, Matsue Seikyo Hospital, Matsue, JPN; 3 Community Care, Unnan City Hospital, Unnan, JPN; 4 Community Medicine Management, Shimane University Faculty of Medicine, Izumo, JPN

**Keywords:** intestinal perforation, capillary leak syndrome, rheumatoid vasculitis, rural hospital, general medicine

## Abstract

Rheumatoid vasculitis (RV) is a rare disease associated with rheumatoid arthritis (RA). The incidence of RV has decreased with the development of treatment for RA. However, some patients still develop RV in rural areas, where medical care for autoimmune diseases is inadequate. In this report, we describe a case of RV complicated by an acute exacerbation of generalized ulcerative lesions and capillary leak syndrome in an 86-year-old woman with a severe joint deformity due to RA. RV is a systemic vasculitis characterized by various symptoms. When a patient with RA is diagnosed with poorly controlled joint deformities, general physicians should consider the possibility of RV. Urgent investigation and intensive treatment should be initiated for vasculitis to support the lives of older patients with advanced RA.

## Introduction

Rheumatoid vasculitis (RV) is a disease that causes inflammation of the blood vessels owing to the deposition of autoantibodies and immune complexes that target the vessel wall [1]. It occurs in patients with long-term severe rheumatoid arthritis (RA) [2]. In many cases, treatment of RV requires intensive immunosuppressive therapy. It results in significantly higher mortality than RA, which, like any other systemic vasculitis, can cause necrosis, vascular occlusion, and tissue ischemia [3].

With recent improvements in the treatment of RA, the progression of joint destruction and transformation to malignant RA is rare [4]. However, rural areas with inadequate autoimmune disease therapy may likely have more patients with RV complicating poorly controlled RA [5-7]. In this report, we describe a case in which a patient with poorly controlled RA developed RV complicated by skin ulcers and intestinal ischemia, and we discuss the future of autoimmune disease treatment in rural areas.

## Case presentation

An 86-year-old woman presented to our clinic with chief complaints of impaired consciousness and pain in the left proximal lower leg. She lived alone in a rural area and was independent without support from her neighbors. One day before the presentation, the patient fell on the dirty floor of her home and became immobile. On the day of the presentation, the patient had presented to our clinic two days previously, having been found collapsed at home by a family member. Her medical history included RA, osteoarthritis, and hypertension. Medication history included methotrexate of 4 mg/week, folic acid, cilnidipine, and bisoprolol fumarate tablets.

**Figure 1:**
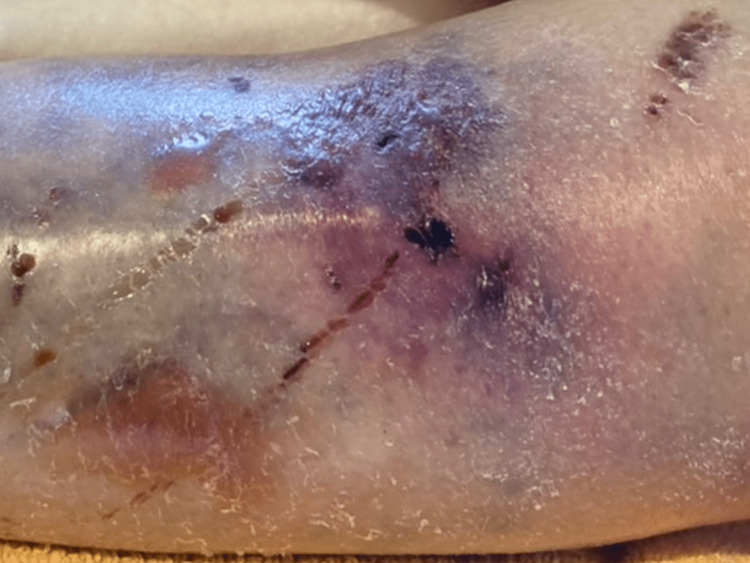
An open fracture of the left lower leg with soft tissue infection

She was alert to the place, person, and time in the initial medical examination. Her height was 1.50 m, and her weight was 48 kg. Her vital signs were as follows: blood pressure of 106/60 mmHg; pulse rate of 114 beats/min; respiratory rate of 38/min; O2 saturation of 100% (10 L/min; mask with reservoir); and temperature of 37.8°C. She became severely dehydrated with a dry mouth and axilla. Her chest and abdominal examinations were normal. The neurological examinations did not show any abnormalities. Her hands and feet joints were severely deformed with RA. Her laboratory evaluations showed severe renal failure with a blood urea nitrogen of 71.7 mg/dL and creatinine of 3.18 mg/dL. The blood culture and wound culture were obtained in the initial presentation. In addition, an open fracture of the left lower extremity caused soft tissue infection as a route of infection and caused sepsis (Figure 1).

The patient was in a state of metabolic acidosis, with a pH of 7.302, because of renal failure and sepsis (Table 1).

**Table 1:** Laboratory data of the patient at admission PR3-ANCA: proteinase 3-antineutrophil cytoplasmic antibody; MPO-ANCA: myeloperoxidase-antineutrophil cytoplasmic antibody; ACPA: anti-cyclic citrullinated peptide antibody; PO2: oxygen partial pressure; PCO2: carbon dioxide partial pressure.

Parameters	Level	Reference
White blood cells	17.4	3.5-9.1 × 10^3 ^/μL
Neutrophils	88.9	44.0-72.0%
Hemoglobin	10.7	11.3-15.2 g/dL
Platelets	29.1	13.0-36.9 × 10^4^/μL
Total protein	7.3	6.5-8.3 g/dL
Albumin	3.7	3.8-5.3 g/dL
Total bilirubin	0.8	0.2-1.2 mg/dL
Aspartate aminotransferase	79	8-38 IU/L
Alanine aminotransferase	36	4-43 IU/L
Uric acid	10.5	3.0-6.9 mg/dL
Blood urea nitrogen	71.7	8-20 mg/dL
Creatinine	3.18	0.40-1.10 mg/dL
Estimated glomerular filtration rate	11.2	>60.0 mL/min/L
Serum sodium	128	135-150 mEq/L
Serum potassium	4.4	3.5-5.3 mEq/L
Serum chloride	86	98-110 mEq/L
Creatine kinase	1984	43-165 U/L
C-reactive protein	8.54	<0.30 mg/dL
Immunoglobulin G	683	870-1700 mg/dL
Immunoglobulin A	242	110-410 mg/dL
Immunoglobulin M	210	35-220 mg/dL
Immunoglobulin E	167	<173 IU/dL
Rheumatoid factor	150	<15 IU/mL
Ferritin	207.1	4.1-120.2 ng/mL
Antinuclear antibodies	<40	<40
Complement 3	82	86-160 mg/dL
Complement 4	8	17-45 mg/dL
PR3-ANCA	<1.0	<3.5 U/mL
MPO-ANCA	<1.0	<3.5 U/mL
ACPA	1.9	<5 U/mL
pH	7.302	
PO_2_	215	80-100 mmHg
PCO_2_	11.3	35.0-45.0 mmHg
Bicarbonate	11.3	20.0-26.0 mmHg
Lactate	7.7	0.56-1.39 mmol/L
Urine leukocyte esterase	Negative	Negative
Urine protein	2+	Negative
Urine blood	Negative	Negative

Considering sepsis, dehydration, and rhabdomyolysis causing acute renal failure, initial treatment consisted of meropenem, intravenous fluids, and continuous hemodiafiltration dialysis. We controlled her foot pain with acetaminophen because of renal failure. Although the patient's general condition improved with the initial treatment, and C-reactive protein decreased to 4.4 mg/dL, generalized edema and a rapid decrease in albumin level could not be explained by increased vascular permeability due to sepsis. We also observed intravascular dehydration and pleural effusion and considered the patient to have capillary leak syndrome secondary to sepsis. Albumin replacement was initiated on the seventh hospital day. We treated the capillary leak syndrome with methylprednisolone 1,000 mg for three days and globulin administration of 4 mg/kg for five days, but the improvement was inadequate.

**Figure 2:**
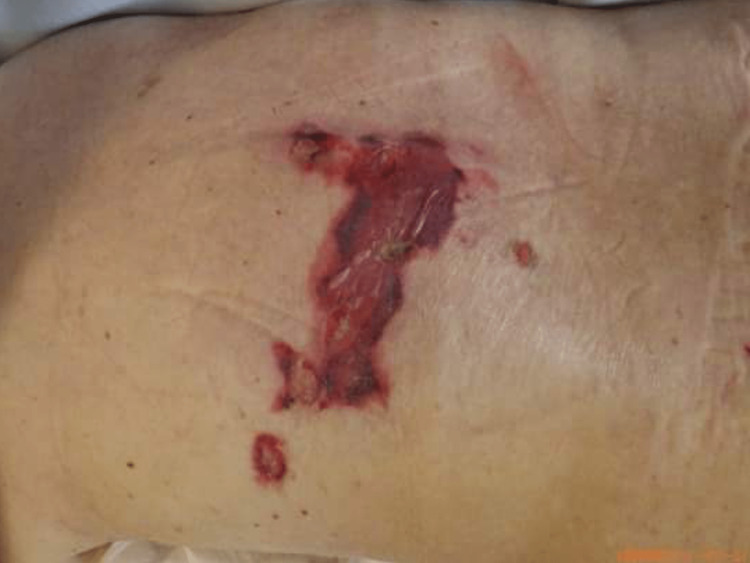
Purpura and ulcers on the back

**Figure 3:**
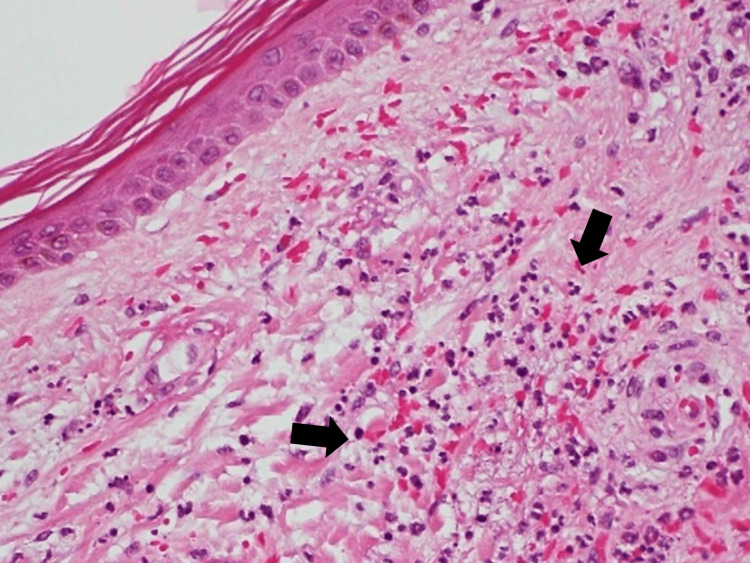
Skin biopsy. Hematoxylin and eosin stain showing necrotizing vasculitis Pathology findings include inflammatory cell infiltrates, such as polymorphonuclear leukocytes and edema in some areas of the shallow dermis, and fibrin deposition and inflammatory cell infiltration in some small vessels' walls (black arrows).

On the ninth hospital day, we conducted a skin biopsy of the purpura and ulcers on the back to identify the cause of capillary leak syndrome (Figure 2). Histopathological examination of the skin revealed active vasculitis (Figure 3).

Because the patient had a poorly controlled RA, a diagnosis of RV was made. Cyclophosphamide 500 mg was administered the next day intravenously for RV, but the inflammatory response remained elevated. Fifteen days later, the patient developed abdominal distention, and an abdominal computed tomography (CT) scan was performed.

On the 15th day of hospitalization, the patient developed sudden hypotension and peripheral circulatory failure after the enterography with the ileus tube to investigate the movement of intestinal tracts. In addition, there was an exacerbation of capillary leak syndrome due to RV and sepsis, which aggravated her hemodynamic status. There may have been distribution shock due to increased intestinal circulation and decreased circulating plasma volume. As dehydration with diuretics limited edema reduction accompanied by low blood pressure under systolic blood pressure of 90 mmHg, we initiated continuous daily albumin administration for hypoalbuminemia to improve the circulating plasma volume by increasing plasma aspiration into the vessels to save the patient's life.

**Figure 4:**
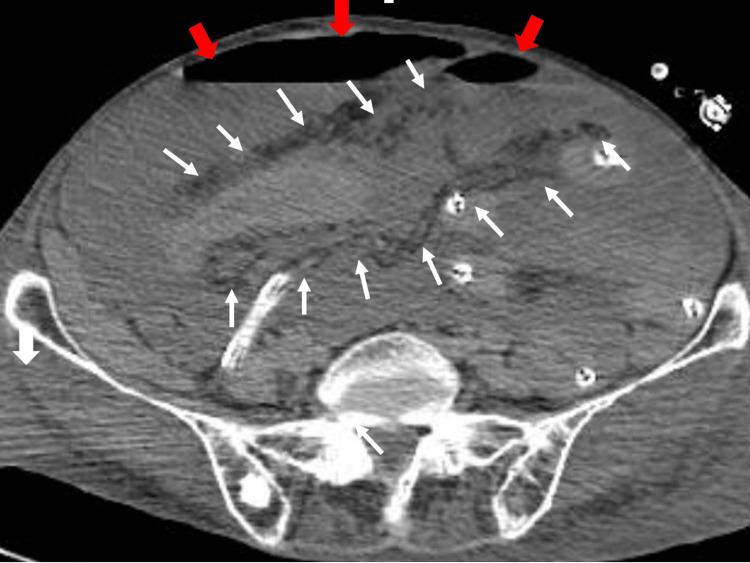
Pelvic computed tomography showing free air just below the abdominal wall (red arrows) and between the small intestine (white arrows)

On the 25th day of hospitalization, we performed abdominal and pelvic CT with contrast to evaluate the abdomen and found free air (Figure 4).

The surgeons performed emergency surgery on the same day. Because of intestinal tract necrosis, they performed a colostomy on the right side of the abdomen with the resection of the ileum partially. Blood pressure recovered, and oxygenation improved. Systemic edema improved dramatically, and C-reactive protein levels decreased despite the surgery. On the 35th day of illness, there was a sudden decrease in blood pressure, and blood cultures were positive for Stenotrophomonas multiformis. The patient was treated with Minomycin (200 mg/day); however, sepsis progressed, and she died on the 40th day of hospitalization.

## Discussion

This case report describes a patient with poorly controlled RA who developed RV, causing capillary leak syndrome and intestinal ischemia. In this case, we found how capillary leak syndrome and intestinal ischemia complicated RV, and hence, it is essential for general physicians to meticulously investigate severe exacerbations of RA and vasculitis at an early stage.

This case suggests that RV may be associated with capillary leakage syndrome. According to the literature, capillary protein permeability is increased in various inflammatory diseases, resulting in fluid leakage from the vessels into the interstitium [8,9]. Sepsis is the disease that is most commonly associated with capillary leak syndrome [8,9]. However, many other diseases can also present with diffuse indurated edema, exudative serous leakage, non-cardiogenic pulmonary edema, hypotension, and sometimes hypovolemic shock with multiple organ failure [8,9]. Capillary leak syndrome is the term used to describe this set of conditions associated with increased capillary protein permeability [10]. In our case, there was generalized edema, increased pleural effusion, and a rapid decrease in the albumin level initially thought to be a capillary leak due to sepsis. However, even after the sepsis improved with antibiotics, albumin did not stop leaking out of the blood vessels; therefore, we thought it was capillary leakage syndrome associated with RV, and we initiated RV treatment. In the future, when treating patients with RA, we need to be aware of the possibility of RV with attendant capillary leak syndrome.

Because edema in capillary leak syndrome is systemic, it affects most organs. A case of idiopathic systemic capillary leak syndrome in which intestinal edema was confirmed has been reported [11]. In this case, the paralytic ileus that developed may have been due to restricted intestinal motility caused by intestinal edema. The gastrointestinal manifestations of RV include ischemia, ulceration leading to perforation, and arteritis of the liver, spleen, and pancreas [12]. In this case, there was no apparent thrombus or vasculitis in the resected intestinal lesion, and direct intestinal ischemia due to RV was ruled out. However, vasculitis could have caused indirect intestinal necrosis and subsequent intestinal perforation due to the partial narrowing of blood vessels, limiting blood flow to the intestinal tract. RV causes vasculitis throughout the body [12]. Therefore, when inflammatory findings and abdominal symptoms persist, as in this case, the possibility of intra-abdominal inflammation and intestinal ischemia due to vascular destruction should be considered and promptly addressed.

Rural areas are aging, so RA has increased among older patients and requires comprehensive management in family medicine. In rural areas worldwide, a shortage of rheumatologists results in delayed or inadequate treatment of RA [13]. The progressed RA may not be controlled effectively, impinging on the patient’s quality of life and causing various complications, including RV, as in this case [14]. In rural contexts, the education of family physicians has been provided in Japan to approach multimorbidity in older patients including RA [15]. As specialists in systems, family physicians can deal with complicated medical issues, including RA, in older patients [16]. RA management has been established based on scientific guidelines from international authorities [17,18]. In family medicine education, RA management can be driven more, and education can improve the quality of care for older patients with rheumatic diseases in rural contexts.

## Conclusions

The development of RV is rare, but its course can be severe and fatal when complicated by capillary leak syndrome. During the chronic course of RA, patients with increased systemic inflammation should be treated with immunosuppressive therapy at an early stage to suppress the development of vasculitis symptoms. With the aging population and increasing number of RA patients, family physicians working in rural contexts must constantly scrutinize and treat it with this possibility in mind.
